# CBTp for Schizophrenic and Schizoaffective Patients in a Forensic Psychiatric Setting: A Retrospective Audit

**DOI:** 10.1192/bjo.2022.430

**Published:** 2022-06-20

**Authors:** Patrick Briggs

**Affiliations:** Mersey Care NHS Foundation Trust, Liverpool, United Kingdom

## Abstract

**Aims:**

CBTp is a clinically validated treatment for psychosis with meta-analyses showing beneficial effects for both positive and negative symptoms. CBTp is recommended by NICE for treatment of schizophrenia and psychosis. The aims of this audit were (1) To determine whether patients with schizophrenia or schizoaffective disorder had been offered CBTp as part of their treatment. (2) To determine if patient who were offered CBTp completed the recommended 16 minimum sessions. (3) To identify barriers to the offering and completion of CBTp. (4) Based on the audit findings, provide recommendations to assist in the utilisation of CBTp in the forensic psychiatric setting.

**Methods:**

A retrospective audit was carried out on 30 patients aged 18 years and older from a medium security forensic hospital, Liverpool UK. Patients included had a diagnosis of schizophrenia (F20) or schizoaffective disorder (F25). 26 male patients and 4 female patients were included in the audit, who were inpatients between 01/01/21 and 01/01/22.

Data regarding the offering and completion of CBTp was collected from the electronic health system records and cross-referenced with the psychology team's internal data collection system to ensure that aims (1) and (2) could accurately be assessed and compared with NICE recommendations. Barriers to the offering and completion of CBTp were also documented and categorised into specific groups, with recommendations based on these findings being provided.

**Results:**

The audit found that 68% (19/28) of patients were offered CBTp, with 85% (11/13) of these patients going on to complete the recommended 16 minimum sessions of CBTp. Barriers to the offering of CBT included patients not being mentally well enough of psychological therapies (7/9) and being engaged in other psychological therapies (2/9). The barrier towards completion of 16 sessions of CBTp was patient refusal (2/2).

**Conclusion:**

Implementation of CBTp for all patients with schizophrenia or schizoaffective disorder fell below NICE recommendations that all patients with psychosis should be offered CBTp and completed for at least 16 sessions. However, improvements have been made from previous similar studies, demonstrating a positive trend towards greater levels of psychotherapeutic interventions with schizophrenic and schizoaffective patients. Appropriate reasons for non-compliance were identified for all patients who were not offered CBTp and patient refusal was identified as an obvious barrier to CBTp completion. A framework for implementation will be recommended with an aim to improve patient compliance and overall health outcomes.

